# Effects of Climatic Factors and Disturbance on Plant Above‐Ground Biomass and Species Diversity of Chinese Grasslands: A Meta‐Analysis

**DOI:** 10.1002/ece3.74320

**Published:** 2026-09-08

**Authors:** Wenjie Huang, Yuanyuan Yin, Wei Wang, Fangzheng Liu, Yilei Zhao, Junsheng Li

**Affiliations:** ^1^ Teachers College Beijing Union University Beijing China; ^2^ Institute of Science and Technology Education Beijing Union University Beijing China; ^3^ Institute of Ecology Chinese Research Academy of Environmental Sciences Beijing China; ^4^ Command Center for Nature Resources Comprehensive Survey China Geological Survey Beijing China

**Keywords:** above‐ground biomass, biodiversity, China, climatic factors, disturbance, grassland

## Abstract

Grassland ecosystems provide substantial social and cultural value but are vulnerable to climate change and human activities. Large‐scale evidence on how climate and disturbance jointly affect above‐ground biomass (AGB), species richness, and their relationship remains limited. We conducted a meta‐analysis by compiling and standardizing plot‐level observations from 226 studies of Chinese grasslands. The final database contained 1192 records from 378 sites surveyed between 1982 and 2019, with matched climatic variables (mean annual temperature, annual precipitation, and radiation) and disturbance categories (grazing, mowing, and fencing). Random‐forest models identified disturbance, precipitation, and radiation as the strongest predictors of AGB, whereas temperature was the strongest predictor of species richness. AGB was higher under mowing and fencing than under grazing and declined with increasing grazing intensity; it also increased with local precipitation. Species richness was highest under mowing, followed by fencing and grazing, and was greatest under wetter conditions and moderate temperatures. Across the national dataset, AGB and species richness were linearly and positively related overall, although several plant formations showed a unimodal pattern. Heavy grazing weakened the linear relationship, whereas moderate grazing strengthened it. Our study provides a scientific basis for the management of grassland ecosystems under the influence of climate change and human activities.

## Graphical abstract

This meta‐analysis across China reveals that grassland biomass is primarily driven by human disturbance and precipitation, while biodiversity is more influenced by climate. Both metrics perform better under mowing and fencing than grazing, with biodiversity and biomass generally sharing a positive linear relationship. However, this relationship is weakened by heavy grazing but enhanced by moderate grazing.

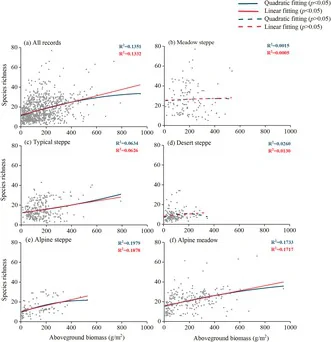

## 1 Introduction

Grasslands are one of the most important ecosystems on the land surface, accounting for 30%–40% of the Earth's surface and 34% of terrestrial carbon stocks (White et al. 2000). Grasslands provide important ecosystem services such as provisioning services, climate regulation, and tourism services (le Provost et al. 2023; Richter et al. 2024). These ecosystem services depend on the biodiversity and biomass production of grasslands (Zhang et al. 2021). In the context of climate change and the increasing intensity of disturbances, biodiversity and productivity are declining in grasslands (Chen et al. 2024; Sun et al. 2022; Zhang, Delgado‐Baquerizo, et al. 2023). Investigating changes in grassland biodiversity and biomass and their natural and anthropogenic drivers could help guide grassland ecosystem management, especially under the background of climate change.

In recent decades, many studies have found that climatic conditions and disturbances are both important factors affecting the GPP or summer above‐ground biomass (AGB) of grassland ecosystems. Precipitation plays an important role in grassland GPP. Increased precipitation leads to a significant increase in grassland GPP (Liang et al. 2017), while decreased precipitation in late summer could lead to a decline in grassland AGB (Brookshire and Weaver 2015). The effects of temperature on grassland GPP showed regional differences. The results of artificial warming experiments showed that the increasing effect of warming on GPP and NPP of alpine grassland was more significant than that of temperate grassland (Wang, Cadotte, et al. 2019). However, in the alpine meadow of the Tibetan Plateau, warming had no significant effect on NPP because warming led to increased below‐ground biomass and decreased AGB (Liu et al. 2018). In arid and semiarid regions, increased warming may lead to reduced soil moisture and thus decrease grassland GPP and AGB (Querejeta et al. 2021). In addition to precipitation and temperature, increased radiation intensity, CO_2_ concentration, and N deposition could enhance the GPP of grassland (Brookshire and Weaver 2015; Liang et al. 2017). In addition to climatic conditions, grassland productivity was closely related to human activities. Results of a 5‐year experiment in a typical steppe on the Loess Plateau of China showed that grazing reduced the above‐ground biomass of grasslands (Zhang, Jin, et al. 2023). A study of a 15‐year fencing experiment in a desert steppe found that fencing increased the AGB (Wu et al. 2019). In grasslands that were simultaneously fertilized and grazed, there was no decrease in above‐ground biomass, as the additional increase in NPP from fertilization was offset by herbivore consumption (Gruner et al. 2008; LeBauer and Treseder 2008). Overall, recent studies have revealed the role of climatic factors and disturbances in driving grassland productivity on individual sites or regions, but there is no unanimous consensus on the dominant factors driving the spatial differences in grassland productivity.

How climate change and disturbances drive biodiversity change are hot issues in ecological research (Fitzpatrick et al. 2018; Pugnaire et al. 2019; van der Putten et al. 2016). There are regional differences in the effects of temperature on grassland biodiversity (Li, Chang, et al. 2024). For example, studies on grasslands in northern China and alpine meadows in the European Rocky Mountains showed that plant diversity was negatively correlated with mean annual temperature because warmer temperatures advance the reproductive phenology of plants, decrease seed production, and thus drastically reduce the soil seed bank, leading to population declines and even localized extinctions (Briceño et al. 2015; Li, Chen, et al. 2024). In alpine meadows in the Rocky Mountains of Colorado, USA, experimental warming removes subterranean seed banks, leading to reduced species richness (Panetta et al. 2018). However, in alpine meadows at higher elevations in Europe, snowpack and permafrost thawing due to warming resulted in a significant increase in plant diversity (Steinbauer et al. 2018). Besides temperature, plant diversity is positively correlated with annual precipitation in grasslands (Li, Chen, et al. 2024). For example, the decrease in winter precipitation from 2000 to 2014 was responsible for the decline in grassland biodiversity in California, USA (Harrison et al. 2015). Furthermore, many studies have shown that disturbances are also an important driver affecting grassland biodiversity (Harrison et al. 2020; Luo et al. 2023; Zhu et al. 2021). Grazing and mowing increase plant diversity by regulating the density, height, and number of dominant species because accompanying changes in the availability of light resources increase the number of annual herbs (Zhang, Zhang, et al. 2023). The effect of grazing on grassland biodiversity may depend on the degree of drought in the grassland. Grazing had less effect on biodiversity in meadow steppe with better moisture conditions, but in more arid typical steppe and desert steppe, grazing significantly reduced biodiversity (Zhang, Zhang, et al. 2023). Fencing could reduce the species richness of grassland communities (Adler and Bradford 2002; Saatkamp et al. 2018). However, recent studies have found that the effect of fencing on grassland biodiversity was related to the number of years of fencing. For example, plant diversity in a Chinese meadow steppe first decreased and then increased when the fencing period reached 11 years (Gao et al. 2021).

AGB and plant species richness in grasslands often show a specific relationship. Several studies have found a significant positive correlation between them, i.e., communities with high productivity have higher biodiversity (Bai et al. 2007; Gillman and Wright 2006). However, other studies found an unimodal relationship, i.e., when productivity is high to a certain extent, competitive interspecific effects lead to lower community biodiversity (Chase et al. 2018; Chase and Knight 2013; Fraser et al. 2015; Pärtel et al. 2007). A study pointed out that there was no clear relationship between productivity and fine‐scale (m^−2^) richness within sites, within regions, or across the globe (Adler et al. 2011). Grazing may alter relationships between AGB and diversity (Cao et al. 2024). For example, a 5‐year experiment in a typical steppe of the Loess Plateau in China found that the relationship between plant species and AGB was negatively linear in the ungrazed condition, but a hump‐shaped relationship was found in the grazed condition (Zhang, Zhang, et al. 2023). A study of meadow steppe in the Han River Plain, China, found that plant diversity and productivity were positively correlated under low‐ and medium‐intensity grazing, but negatively correlated under high‐intensity grazing (Wei et al. 2023). Thus, the relationship between grassland productivity and diversity is still under debate.

Overall, existing studies have revealed the effects of climatic factors on grassland biomass and biodiversity, while the effects of disturbances have been relatively poorly recognized. The relationship between plant diversity and productivity in grasslands is still controversial and needs to be examined using large‐scale observational data. By integrating observations across multiple sites and grassland types, large‐scale syntheses can distinguish broadly consistent patterns from site‐specific responses caused by spatial heterogeneity. Because China spans a wide geographical range and encompasses diverse grassland types, it provides an ideal setting for such a large‐scale synthesis. Therefore, we chose Chinese grassland as the study area and conducted a meta‐analysis using AGB and species‐richness data from 1192 plot‐level records at 378 sites. We first identified anthropogenic and climatic factors affecting the AGB of different grassland types in the past 30 years using a random forest model. Second, we analyzed the differences in the relationship between AGB and species richness in various grassland types as well as under various grazing intensities. This paper aimed to reveal (1) the effects of climatic factors and grazing intensity on AGB and species richness and (2) the effects of grazing intensity on the relationship between AGB and species richness of grasslands in China.

## 2 Materials and Methods

### 2.1 Study Area

**FIGURE 1:**
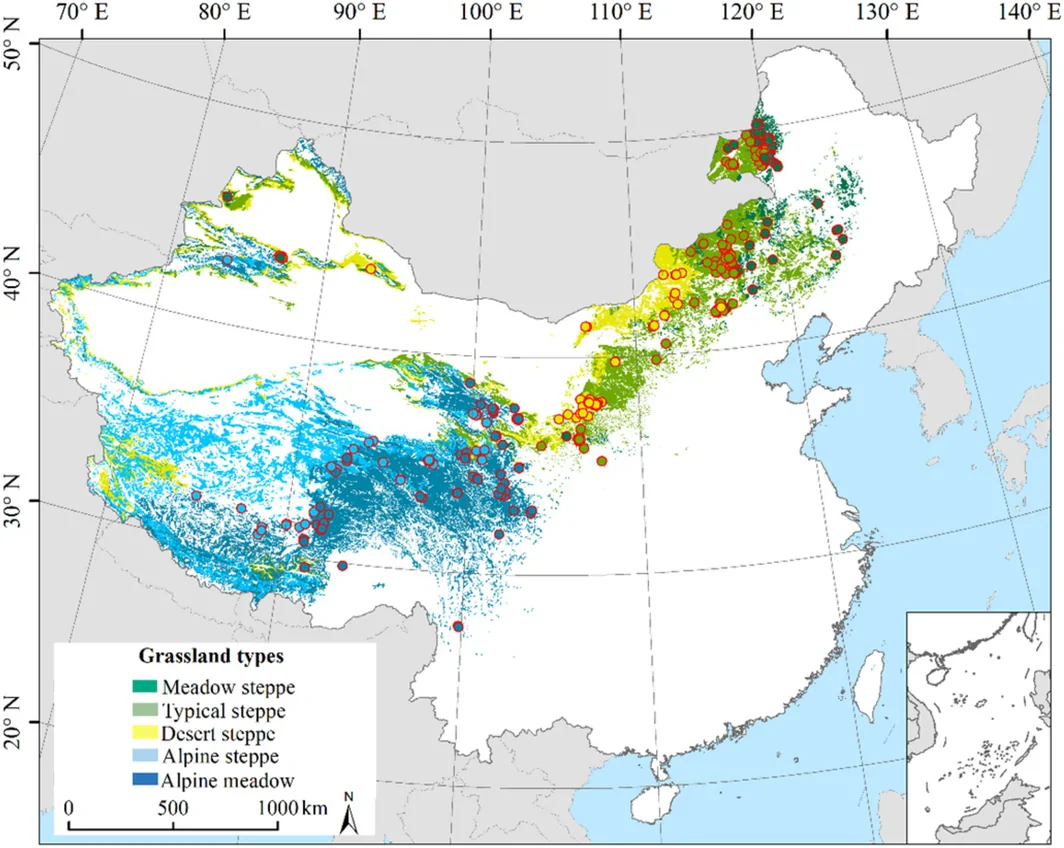
Spatial distribution of 378 sites in the meta‐analysis of grassland above‐ground biomass and species richness.

Grasslands in China are mainly distributed in the Mongolian Plateau in the north and the Tibetan Plateau in the southwest. According to the vegetation map of China (Editorial Committee of China Vegetation Map 2007), grassland types in China are mainly divided into five types, which are meadow steppe, typical steppe, desert steppe, alpine meadow, and alpine grassland, in order from northeast to southwest (Figure 1). These grassland types have different climatic characteristics and constructive species (Table 1). Alpine meadows have the highest precipitation and lowest temperature, and the main constructive species include *Kobresia humilis and Kobresia tibetica*. Desert steppe has the lowest precipitation and highest temperature, and its constructive species are *Stipa capillata* and *Stipa tianschanica* .

**TABLE 1:** Climate and community structure of different types of grassland in China.

Grassland type	Constructive species	T_mean_ (°C)	*p*_mean_ (mm)
Meadow steppe	*Stipa baicalensis, Leymus chinensis*	1.2	429.6
Typical steppe	*Stipa grandis, Leymus chinensis, Stipa krylovii, Stipa bungeana*	2.5	339.3
Desert steppe	*Stipa capillata, Stipa tianschanica, Stipa breviflora*	3.5	250.5
Alpine steppe	*Stipa purpurea*	−3.9	343.5
Alpine meadow	*Kobresia humilis, Kobresia pugmaea, Kobresia capillifolia*	−1.8	511.7

Abbreviations: *p*_mean_, Annual total precipitation averaged from the respective distribution range; *T*_mean_, mean annual temperature averaged from the respective distribution range.

### 2.2 Data

In this study, we searched using the following keywords: “China”, “Qinghai‐Tibetan Plateau”, “Qinghai”, “Tibet”, “Xinjiang”, “Inner Mongolia”, “alpine steppe”, “alpine meadow”, “grassland”, “herbaceous plants”, “productivity”, “above‐ground biomass”, “species richness”, “biodiversity” from China National Knowledge Infrastructure (CNKI) (http://www.cnki.net/) and Web of Science (https://www.webofscience.com/), respectively. The literature and data‐screening workflow is summarized in Figure S1. After bibliographic and source reconciliation and the record‐overlap audit, 226 unique studies were retained. From these studies, we extracted specific survey information, including the name of the site surveyed, the observation year, location (latitude and longitude, and elevation), grassland type, constructive species, plot size, the number of quadrats in each plot, type of disturbances, AGB, and species richness (number of plant species). For each site, several plots were surveyed, and several quadrats were sampled from each plot (usually 1 × 1 m). For each plot, the reported AGB and species richness were averaged from all quadrats within each plot. AGB values were converted to g m^−2^; species richness was retained as the number of plant species per plot.

According to the original author's description, we classified the types of disturbances into fencing, mowing, light grazing, moderate grazing, and heavy grazing. Fencing could avoid herbivore grazing and was extensively used to restore degraded grassland. Mowing was defined as the removal of biomass by clipping vegetation at a height of 10 cm above the soil surface. The mowing frequency was generally once a year, although parts of plots may be mowed twice a year or once every 2 years. Light, moderate, and heavy grazing were determined according to descriptions in the original literature. Because equivalent grazing pressure varies with livestock species and body size, grazing duration, grassland productivity, and local carrying capacity, original literature usually used forage utilization (percent of forage used) to divide grazing intensity. Forage utilization near 30%, 50%, and 70% recurred as reference levels for light, moderate, and heavy grazing, respectively. If the original literature did not report the degree of grazing, we labeled it as unknown.

The preliminary database comprised 1192 records and 378 sites (Table S1). The final response‐specific complete‐case datasets contained 1187 AGB records and 893 species‐richness records; 888 records contained both responses. The time range of the vegetation survey was 1982–2019, with the highest number of records during 1998–2018. Based on the geographic locations of each site and vegetation maps, these sites were identified as five grassland types. Among them, 80 sites were located in the meadow steppe, 128 in the typical steppe, 50 in the desert steppe, 86 in the alpine meadow, and 34 in the alpine steppe. We further determined the plant formation of each plot according to the constructive species. Plots with the same constructive species were grouped into a plant formation.

Regarding the climate data, we used the China Meteorological Forcing (CMF) dataset (Yang and He 2016), which was downloaded from the Cold and Arid Regions Science Data Center of China (http://westdc.westgis.ac.cn/, in Chinese). The dataset covered the period 1979–2015, with a spatial resolution of 0.1° and a temporal resolution of 3 h. This study utilized the near‐surface air temperature, precipitation, and downward shortwave radiation data from this dataset. When analyzing the effects of climatic factors on AGB and biodiversity, the mean annual temperature, annual total precipitation, and mean annual downward shortwave radiation corresponding to each record were extracted according to the location of sites and the year of the survey.

### 2.3 Statistical Analysis

In this study, we used the random forest model to identify the main factors affecting AGB and species richness because this model could accommodate nonlinear relationships between dependent and independent variables and multicollinearity among predictors (Beguería et al. 2014; Gill et al. 2017). The response variable was either AGB or species richness for each plot. The explanatory variables included mean annual temperature, annual total precipitation, mean annual radiation, type of disturbance, and grassland type. The importance of each explanatory variable was determined by the percentage increase in the mean square error (%IncMSE) (De Keersmaecker et al. 2015).

Prior to random‐forest modeling, we assessed predictor collinearity separately for the response‐specific complete‐case datasets. Pearson's correlation coefficients were calculated among the three continuous climatic predictors: mean annual temperature (MAT), mean annual precipitation (MAP), and radiation. Because grassland type and disturbance were nominal categorical predictors, their numerical category codes were not treated as continuous variables. Generalized variance‐inflation factors (GVIFs) were therefore calculated for the complete five‐predictor set, and GVIF^(1/(2df)) was used to compare terms with different degrees of freedom. The maximum predictor |r| values were 0.259 and 0.255, and adjusted GVIF values ranged from 1.016 to 1.695 and from 1.025 to 1.655 (Table 2), respectively, indicating no problematic predictor collinearity.

**TABLE 2:** Collinearity diagnostics for predictors in the random‐forest models.

Generalized variance‐inflation factors					
Response	Predictor	df	GVIF	GVIF^(1/(2df))	Model *n*
AGB	MAT	1	2.346	1.532	1187
	MAP	1	1.901	1.379	1187
	Radiation	1	2.873	1.695	1187
	Grassland type	4	10.411	1.340	1187
	Disturbance	5	1.170	1.016	1187
Species richness	MAT	1	2.463	1.569	893
	MAP	1	1.969	1.403	893
	Radiation	1	2.740	1.655	893
	Grassland type	4	10.296	1.338	893
	Disturbance	5	1.277	1.025	893

Pairwise Pearson correlations among continuous climatic predictors					
Response	MAT–MAP	MAT–radiation	MAP–radiation	Maximum |*r*|	Model *n*
AGB	−0.161	−0.165	0.259	0.259	1187
Species richness	−0.098	−0.211	0.255	0.255	893

*Note:* For predictors with multiple degrees of freedom, GVIF^(1/(2df)) should be used for comparison. Values were calculated from the response‐specific complete‐case datasets used in the final models.

Abbreviations: GVIF, generalized variance‐inflation factor; MAP, mean annual precipitation; MAT, mean annual temperature.

Random‐forest regression models were fitted in R 4.6.1 using the randomForest package version 4.7‐1.2. Hyperparameters were tuned across 15 combinations of mtry (1–5) and terminal‐node size (nodesize = 5, 10, or 20). Each candidate model contained 500 trees and was evaluated using fivefold cross‐validation grouped by publication and repeated five times, so that all records from the same publication were assigned to the same fold. Out‐of‐fold predictions were pooled within each repeat to calculate *R* ^2^, root mean squared error (RMSE), and mean absolute error (MAE), and performance values are reported as the mean ± SD across the five repeats. The combination with the highest mean pooled out‐of‐fold *R* ^2^ was selected, with RMSE as the tie‐breaker. The selected parameters were mtry = 1 and nodesize = 10 for AGB, and mtry = 1 and nodesize = 20 for species richness. Final models contained 2000 trees and used sampling with replacement.

Partial‐dependence functions were calculated while holding the remaining predictors at their observed reference distribution. Pointwise 95% uncertainty intervals were obtained from 500 publication‐cluster bootstrap refits, each containing 500 trees and using the selected hyperparameters. The final 2000‐tree model provided the central partial‐dependence estimate, and the 2.5th and 97.5th percentiles of the bootstrap distribution provided the interval limits.

In order to investigate the relationship between AGB and biodiversity, we fitted their relationship using linear and polynomial fits (Fraser et al. 2015):

(1) y=a+bx

(2) $$y=c+\mathrm{dx}+{\mathrm{ex}}^{2}$$

where *y* represents AGB and *x* represents species richness; *a*, *b*, *c*, *d*, and *e* are fitting parameters.

We first fitted the above equations with all records. Second, the two equations were fitted for different vegetation types and for different types of disturbances. The coefficients of determination (*R* ^2^) of the two equations were compared to determine whether the relationship between AGB and species richness tended to be linear or unimodal.

## 3 Results

### 3.1 Spatial Patterns of AGB and Species Richness in Chinese Grasslands

**FIGURE 2:**
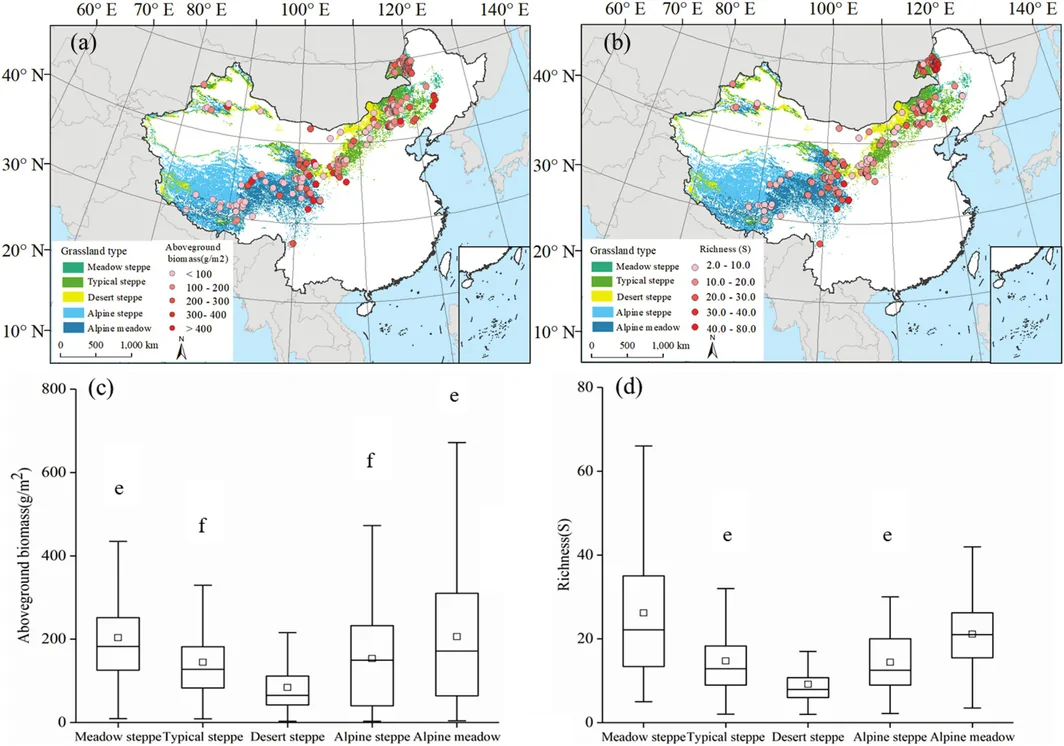
Spatial distribution of above‐ground biomass and species richness in China. (a) Above‐ground biomass at each site; (b) species richness at each site; (c) boxplot of above‐ground biomass for each grassland type; and (d) boxplot of species richness for each grassland type. The same letters represent no significant differences based on Fisher's LSD test. The bands near the middle are the median. Boxes designate the mean value.

We first calculated the site‐level AGB and species richness by averaging data from all plots for each site. AGB and species richness in Chinese grasslands showed obvious regional differences. The areas with higher AGB were mainly distributed in the meadow steppe area in the northeast (203.82 g/m^2^) and the alpine meadow area in the eastern part of the Tibetan Plateau (206.10 g/m^2^), while the desert steppe in the north‐central part of the country had the lowest AGB (84.86 g/m^2^) (Figure 2a). There was no significant difference in AGB between typical steppe and alpine steppe, and no significant difference between alpine meadow and meadow steppe, while a significant difference (*p* < 0.05) in AGB was found between each pair of grassland types (Figure 2c).

The spatial pattern of species richness was similar to that of AGB, with the highest species richness in the meadow steppe (21.13) and alpine meadow (26.18) in the northeast, and the lowest species richness in the desert steppe (9.14) (Figure 2b). There were significant differences in species richness among each pair of grassland types except for typical steppe and alpine steppe (Figure 2d).

### 3.2 Effects of Climatic Factors and Disturbances on AGB

**FIGURE 3:**
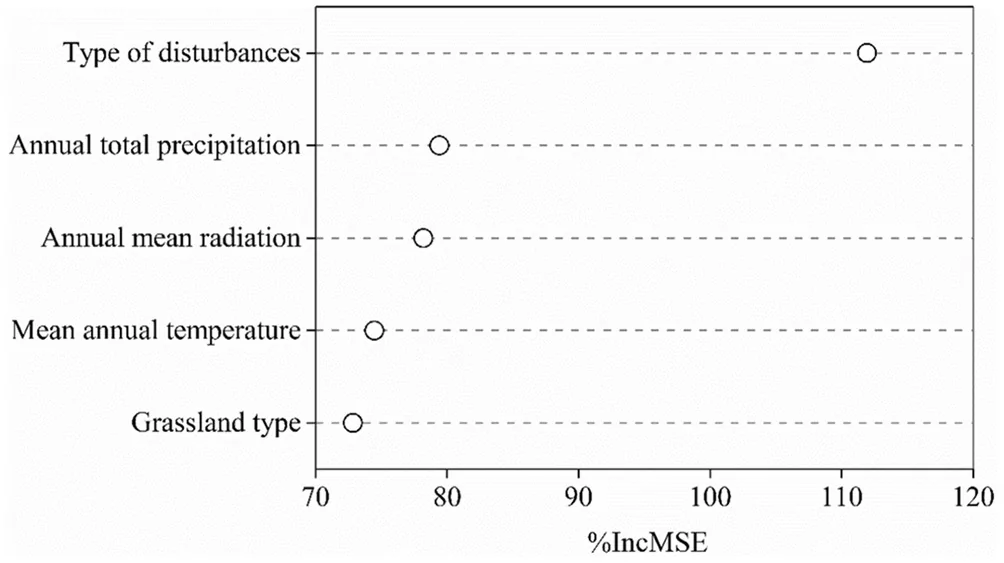
Scaled permutation importance of predictors for AGB in the random‐forest model.

**FIGURE 4:**
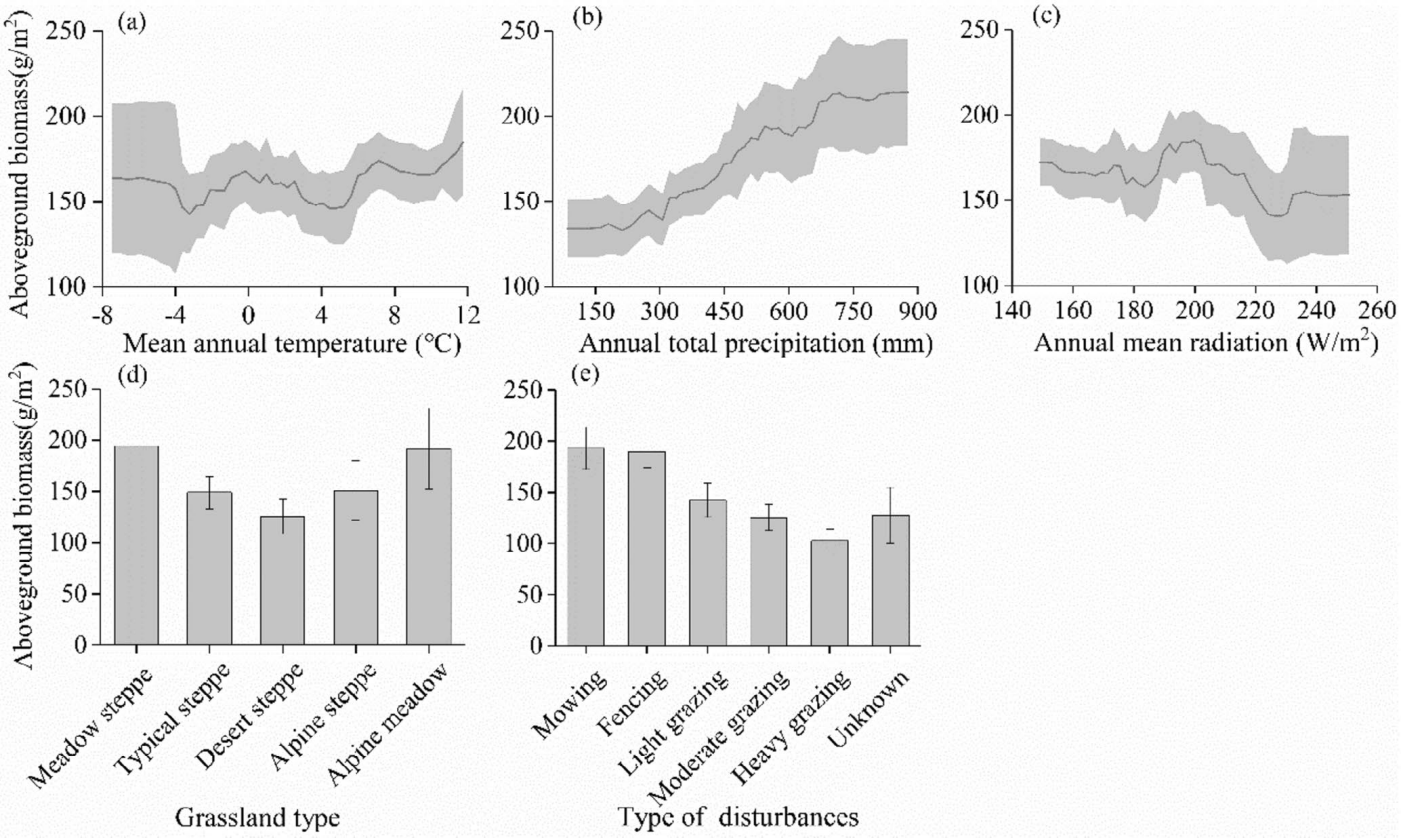
Partial‐dependence responses of AGB to the five predictors. (a) mean annual temperature; (b) annual total precipitation; (c) annual mean radiation; (d) grassland type; (e) type of disturbances. Shaded bands for continuous predictors and error bars for categorical predictors denote pointwise 95% publication‐cluster bootstrap intervals.

Repeated publication‐grouped cross‐validation yielded *R* ^2^ = 0.292 ± 0.018, RMSE = 104.628 ± 1.313 g m^−2^, and MAE = 74.903 ± 1.149 g m^−2^; thus, the model explained approximately 29.2% of the variance in observations from held‐out publications. Disturbance had the highest scaled permutation‐importance score (111.937), followed by MAP (79.425), radiation (78.191), MAT (74.480), and grassland type (72.855) (Figure 3). AGB was greatest under mowing and fencing and decreased with increasing grazing intensity (Figure 4e). It increased almost linearly with precipitation (Figure 4b). AGB peaked near a mean annual radiation of 200 W m^−2^ and declined at lower or higher radiation (Figure 4c), whereas no clear response to MAT was evident (Figure 4a). Among grassland types, AGB was highest in meadow steppe and alpine meadow, intermediate in alpine steppe and typical steppe, and lowest in desert steppe (Figure 4d).

### 3.3 Effects of Climatic Factors and Disturbance on Species Richness

**FIGURE 5:**
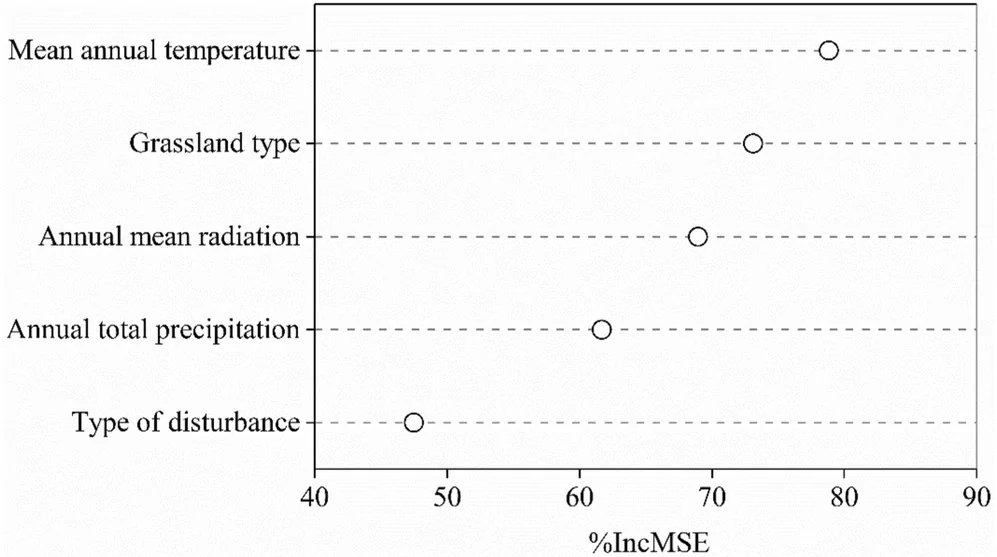
Scaled permutation importance of predictors for species richness in the random‐forest model.

**FIGURE 6:**
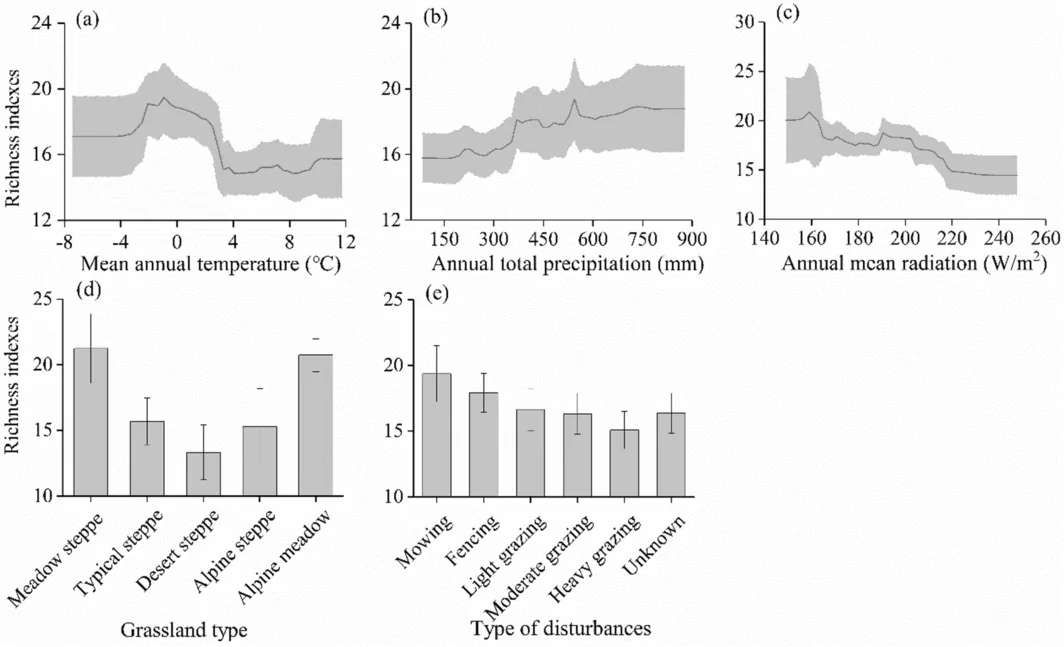
Partial‐dependence responses of species richness to the five predictors. (a) mean annual temperature; (b) annual total precipitation; (c) annual mean radiation; (d) grassland type; (e) type of disturbances. Shaded bands for continuous predictors and error bars for categorical predictors denote pointwise 95% publication‐cluster bootstrap intervals.

Repeated publication‐grouped cross‐validation yielded *R* ^2^ = 0.171 ± 0.025, RMSE = 9.955 ± 0.153, and MAE = 7.144 ± 0.057; thus, the model explained approximately 17.1% of the variance in observations from held‐out publications. MAT had the highest scaled permutation‐importance score (78.834), followed by grassland type (73.111), radiation (68.933), MAP (61.671), and disturbance (47.471) (Figure 5). Species richness was highest at mean annual temperatures of approximately −2°C to 0°C, declined sharply from 0°C to 5°C, and was lowest between 5°C and 10°C (Figure 6a). It was greatest in meadow steppe and alpine meadow and lowest in desert steppe (Figure 6d). Species richness peaked near a mean annual radiation of 160 W m^−2^ and then declined with increasing radiation (Figure 6c), whereas it generally increased with precipitation (Figure 6b). Although disturbance was less important than the climatic predictors, species richness remained highest under mowing and fencing and lowest under heavy grazing (Figure 6e).

### 3.4 Relationship Between AGB and Species Richness for Different Grassland Types

**FIGURE 7:**
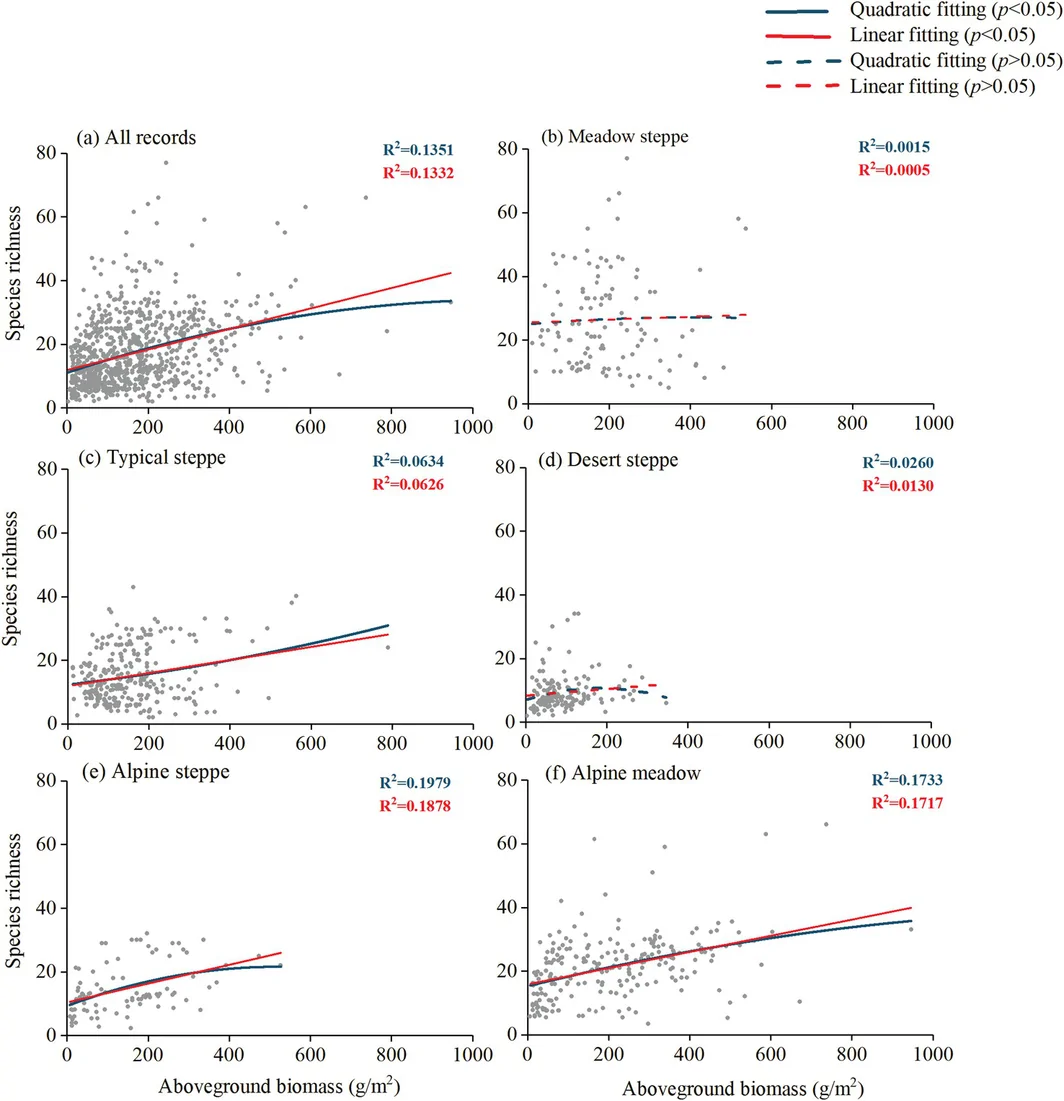
Relationships between above‐ground biomass and species richness in different grassland types. (a) All records; (b) Meadow steppe; (c) Typical steppe; (d) Desert steppe; (e) Alpine steppe; (f) Alpine meadow.

**FIGURE 8:**
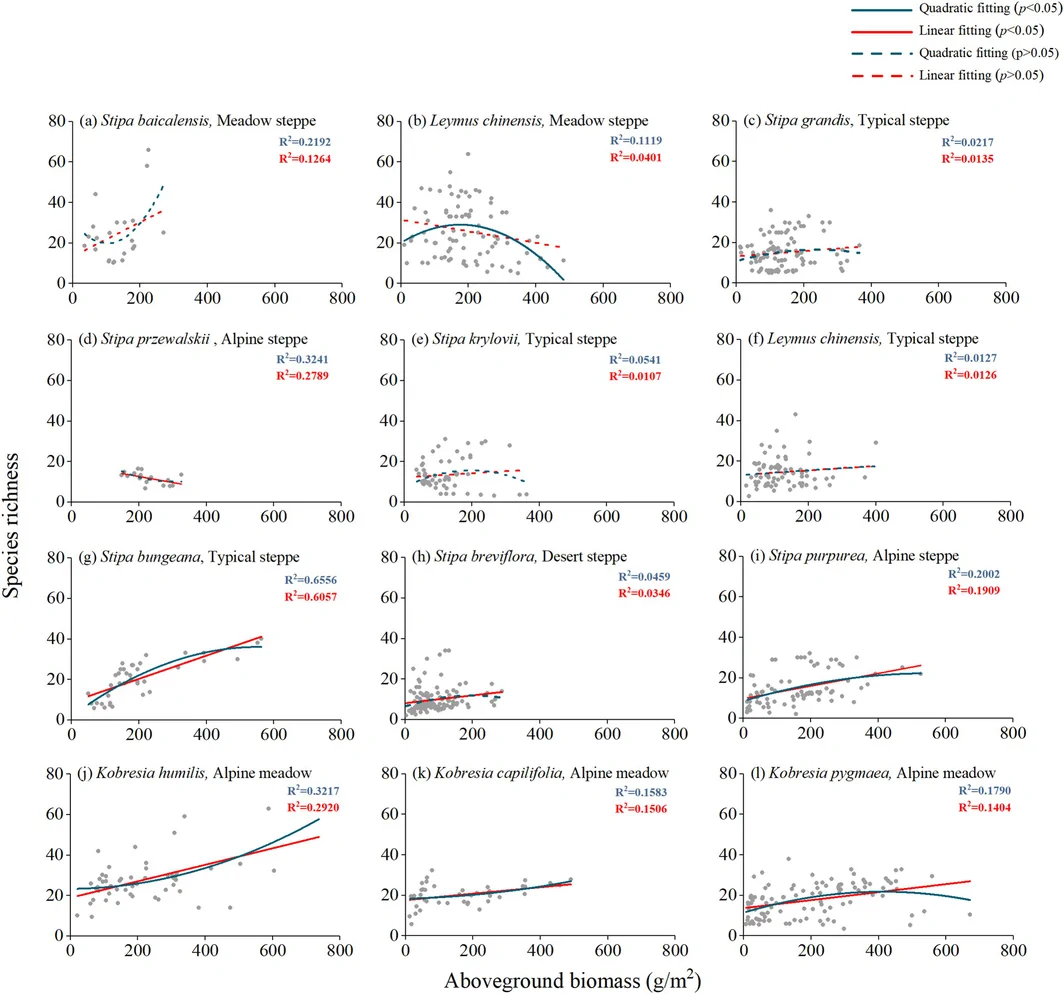
Relationships between above‐ground biomass and species richness in different plant formations. We only analyzed the formation with at least 15 sites. (a) *Stipa baicalensis*, Meadow steppe; (b) *Leymus chinensis*, Meadow steppe; (c) *Stipa grandis*, Typical steppe; (d) *Stipa przewalskii*, Alpine steppe; (e) *Stipa krylovii*, Typical steppe; (f) *Leymus chinensis*, Typical steppe; (g) *Stipa bungeana*, Typical steppe; (h) *Stipa breviflora*, Desert steppe; (i) *Stipa purpurea*, Alpine steppe; (j) *Kobresia humilis*, Alpine meadow; (k) *Kobresia capillifolia*, Alpine meadow; (l) *Kobresia pygmae*a, Alpine meadow.

For all records, there was a significant positive correlation (*p* < 0.05) between species richness and AGB (Figure 7a). The quadratic fit and linear fit showed very similar *R* ^2^, thus, we could conclude that species richness increased linearly with increasing AGB across the study area. For different vegetation types, the correlation between AGB and species richness was not significant in meadow steppe and desert steppe (*p* < 0.05). For other grassland types, AGB and species richness correlated with each other positively and significantly. However, the *R* ^2^ values of quadratic and linear fits were not significantly different, and the quadratic fit did not show any obvious peak points within the data range. Furthermore, we examined the relationship between species richness and above‐ground biomass at a smaller scale (Figure 8). The results showed that 10 formations were dominated by a linear relationship, and only two formations (*Leymus chinensis* meadow steppe and *Kobresia pygmea* alpine meadow) were characterized by a unimodal pattern (*R* ^2^ of the quadratic fit was significantly larger than that of the linear fit). Therefore, differences in the study scale affected the relationship between species richness and AGB.

### 3.5 Relationship Between AGB and Species Richness for Different Types of Disturbances

**FIGURE 9:**
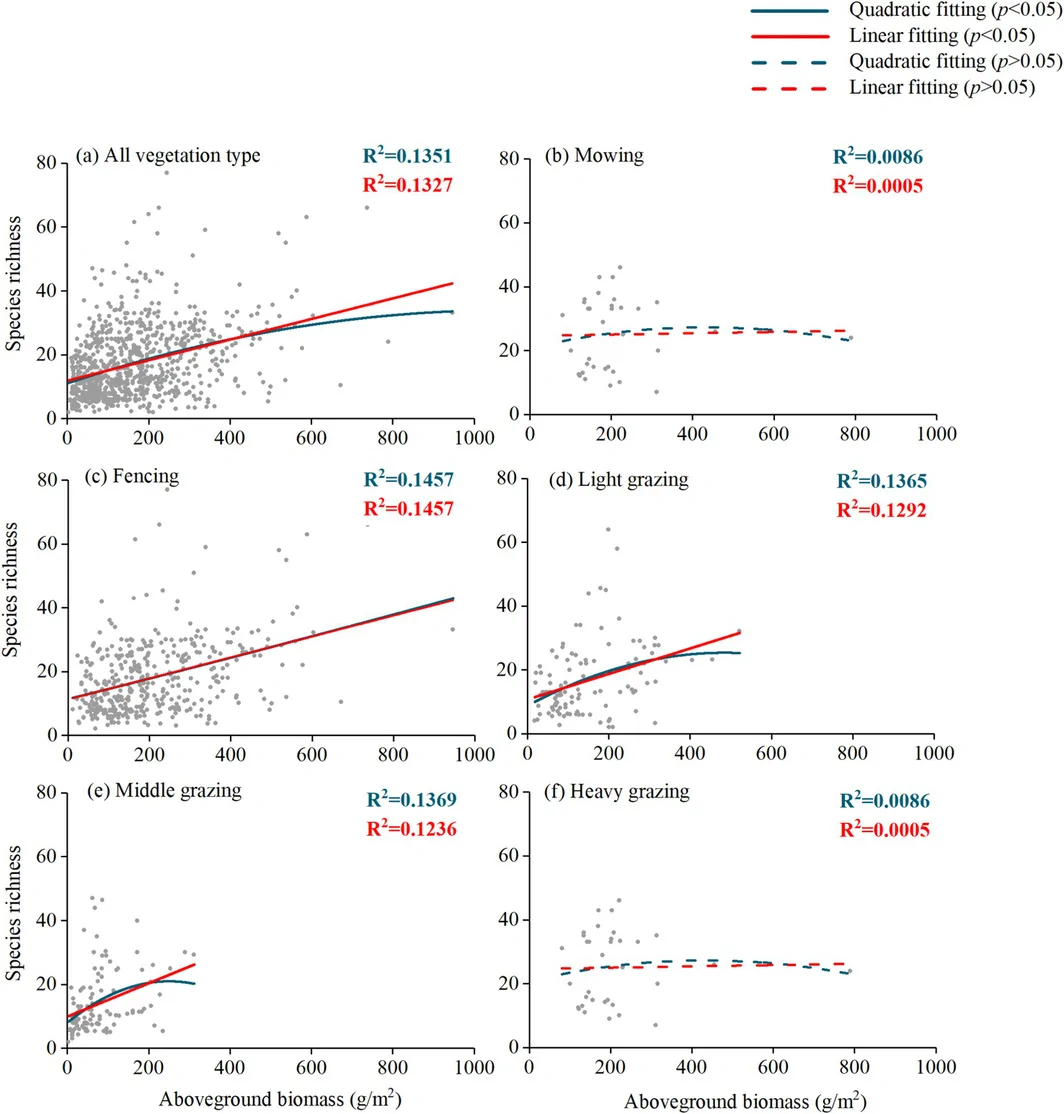
Relationship between above‐ground biomass and species richness for different types of disturbances. (a) All vegetation type; (b) Mowing; (c) Fencing; (d) Light grazing; (e) Middle grazing; (f) Heavy grazing.

We analyzed the effects of different types of disturbances on the relationship between AGB and species richness. The relationship between AGB and species richness was not significant for mowing and heavy grazing, and the *R* ^2^ for both quadratic fit and linear fit was less than 0.01. However, there was a significant positive correlation between species richness and AGB under all other disturbance types. The *R* ^2^ for both fitted curves was similar under fencing (Figure 9c), light grazing (Figure 9d), and moderate grazing (Figure 9e). Meanwhile, the curves of the quadratic fit did not show significant peak points. Thus, the relationship between AGB and species richness was still predominantly linear.

## 4 Discussion

### 4.1 Factors Affecting AGB of Grasslands

We showed that grazing affects the AGB of grassland more than climatic factors. AGB was larger under mowing and fencing, and it decreased with increasing grazing intensity. The effect of grazing on productivity beyond climate change was also confirmed by controlled experiments conducted in alpine meadows on the Tibetan Plateau (Zhang et al. 2015). Since the forage makes up a large proportion of grassland biomass, more AGB is consumed by the livestock under higher grazing intensity. A three‐year survey on grasslands with short‐term fencing (6–8 years) on the Tibetan Plateau found that the AGB of grassland under short‐term fencing was significantly higher than that of grasslands under grazing (Yan and Lu 2015). However, long‐term fencing may result in lower AGB. For example, an experimental study on the desert steppe in northwest China found that AGB was maximized after 11–13 years of fencing, while fencing for 22–24 years resulted in a decrease in AGB because long‐term fencing led to the degradation of vegetation and soil nutrients and the decrease in biodiversity (Liu et al. 2019). As most of the sites extracted in this study were fenced for less than 15 years, fencing still contributed to the increase of AGB.

Besides anthropogenic disturbances, moisture is the most important factor affecting AGB in grasslands. The AGB was greater in areas with higher annual precipitation. When precipitation increases, the increased water available in the soil could be absorbed and utilized by herbaceous plants, promoting their growth and thus enhancing AGB (Knapp et al. 2016). The temperature could affect water availability and plant physiological processes. In the present study, it was found that AGB was highest in the region of moderate temperature, probably because there exists an optimum temperature range for plant growth (Odum 1980). High temperatures increase evapotranspiration and reduce available soil water, which may reduce photosynthetic assimilation and cumulative nutrient uptake by plants during the growing season, leading to reduced AGB (Epstein et al. 1997; Querejeta et al. 2021; Wang et al. 2012). Conversely, in regions of lower temperatures, although evapotranspiration was reduced, low temperatures limited the efficiency of photosynthesis and nutrient utilization by plants, also leading to reduced AGB (Li et al. 2013). Radiation was also a factor modulating the AGB of Chinese grassland. In this study, when the annual mean radiation was 200 W/m^2^, the above‐ground biomass reached its maximum value. In areas with lower or higher radiation, above‐ground biomass decreased. Low radiation may cause a decline in plant photosynthesis efficiency and productivity (Knoh and Baldocchi 2008). High radiation intensifies soil moisture evaporation and damages Photosystem II complex, thus reducing the rate of photosynthesis and productivity (Dong et al. 2016).

### 4.2 Factors Influencing Biodiversity

In this study, temperature had the greatest effect on plant diversity in Chinese grasslands. Species richness was highest near a mean annual temperature of 0°C and declined above or below this threshold (Figure 6a). This unimodal response may reflect water–heat coupling rather than the effect of temperature alone. At low temperatures, thermal constraints limit the number of herbaceous species able to complete their life cycles. At high temperatures, increased evapotranspiration reduces plant‐available soil moisture and intensifies drought stress, thereby excluding drought‐intolerant species (Querejeta et al. 2021). Consequently, the effect of warming on plant diversity likely depends on whether precipitation can compensate for temperature‐driven water loss. In dry and hot environments, rapid species turnover, especially among short‐lived species, may further reduce biodiversity (Cleland et al. 2013).

We found that biodiversity decreased as radiation increased. Radiation is usually not a major limiting factor for photosynthesis in grassland areas, but increased radiation could increase evapotranspiration, which enhances water limitation for plant growth and consequently reduces biodiversity (Kang et al. 2023; Yan et al. 2023). On the other hand, in areas of high solar radiation, ultraviolet radiation may cause damage to plant photosystems, especially affecting the normal functioning of photosystem II, which reduces plant photosynthetic capacity, thus reducing species diversity in grasslands (Ekwealor et al. 2021).

In this paper, we found that species richness gradually increased with increasing precipitation within a certain precipitation range (100–600 mm/a). This is similar to the findings of previous studies. For example, in the Central European steppe, areas with better moisture conditions could simultaneously satisfy the water demand of mesophytic and xerophytic plants, resulting in greater species richness (Cornwell and Grubb 2003). In addition, as precipitation increases, more soil water promotes higher soil microbial abundance, which not only increases the number of plants in symbiotic relationships with microorganisms, but also accelerates the cycling of matter and energy flow, providing nutrients needed for the survival of more organisms, thus increasing species richness (Pan et al. 2024). The results of this study also found that species richness becomes stable when precipitation reaches a certain amount (> 600 mm/a), which was consistent with previous studies. For example, Adler and Levine (2007) found that after a certain level of precipitation, the sustained increase in precipitation did not appear to have a stronger effect on species richness in the central grasslands region of North America. The likely reason is that sufficient precipitation no longer increases soil moisture (Basu et al. 2024; Zhang, Zhang, et al. 2023).

According to the results of this study, disturbances exhibited relatively less effect on grassland biodiversity compared to climatic factors. However, we still found that grassland biodiversity was higher in grasslands under mowing and fencing, and biodiversity decreased with increasing grazing intensity, which is consistent with the findings of Zhang et al. (2016). Disturbances mainly affect biodiversity indirectly by changing species interactions and environmental conditions. Mowing mainly reduced the turnover of dry matter and lowered vegetation height, but did not affect vegetation structural heterogeneity and did not reduce species richness (Smith et al. 2018). Light and moderate grazing had some positive effects on biodiversity. Livestock nibbling can reduce the number of dominant plants, increase competition among species, and expand the ecological niche of the community. At the same time, livestock excreta and trampling can increase soil porosity and introduce more N and P nutrients into the grassland, thus promoting the growth and settlement of more species (Hutchings et al. 2001; Kölbl et al. 2011). Light and moderate grazing could also increase light transmission to the plant community and thus reduce the competition for light among plants (Brown and Whitmore 1992). These factors have positive effects on biodiversity and partially offset the negative effects of grazing. However, under heavy grazing conditions, excessive trampling by livestock can seriously damage soil structure and surface crust, reduce vegetation height and cover, and lead to increased wind erosion of the bare surface, reducing the N and P content of the soil (Li et al. 2007). At the same time, evapotranspiration of the soil is enhanced, and water loss is accelerated, making it difficult for more species to survive and ultimately leading to a significant reduction in biodiversity (Hao and He 2019). Thus, although light and moderate grazing can mitigate the negative impacts of grazing on biodiversity to a certain extent, the effects of heavy grazing cannot be offset.

### 4.3 Relationship Between AGB and Species Richness

In this study, AGB was used as a conservative proxy for grassland productivity. We found that the AGB–species richness relationship was predominantly linear, which is consistent with the results of many studies. For instance, the results of a 24‐year survey of grasslands in Inner Mongolia found that the relationship between biodiversity and productivity was positively linear at different scales (Bai et al. 2004). However, many studies revealed a unimodal productivity‐biodiversity relationship (Fraser et al. 2015; Wang, Quesada, et al. 2019). For the five grassland types investigated in this study, the AGB ranged from 3 to 940 g^−2^. In contrast, the AGB ranged from 2 to 5711 g^−2^ (Fraser et al. 2015). It was possible that our study only included a certain range of productivity gradients, and an unimodal relationship only occurred at larger productivity gradients. Our study found an unimodal relationship for two formations. Thus, scale effects can influence the productivity‐biodiversity relationship (Brun et al. 2019; Chase and Leibold 2002). At smaller scales, factors such as topography, moisture, and slope orientation may determine the productivity‐biodiversity relationship, but at larger scales, factors such as climate and anthropogenic disturbance play a dominant role.

This study found that mowing and heavy grazing significantly weakened the positive AGB–species richness relationship, while the positive relationship was strongest at moderate grazing intensity. A previous study also found that mowing and heavy grazing pressure eliminated the positive correlation between species richness and above‐ground biomass (Zhu et al. 2020). This may be due to the fact that under higher intensities of disturbance, the soil environment deteriorates, which decreases the diversity of the community species and causes the plant dry matter production to be in a state of stress. In contrast, under light or moderate grazing, new ecological niches appeared in the community, and plant interactions were strengthened and dominated by facilitation, thus reinforcing the positive AGB–species richness relationship.

### 4.4 Limitations

This study has some limitations. First, the vegetation surveys used in this study were conducted from 1982 to 2019 by different investigators, and the sampling protocols were not fully consistent among studies. Although most studies used 1 × 1 m quadrats, differences in quadrat size and sampling effort may affect estimates of AGB and species richness. Species richness is strongly dependent on plot area, and comparisons among vegetation surveys with different plot areas require appropriate harmonization (Barandun et al. 2026). Survey date may also affect both variables because the seasonal peaks of AGB and species richness do not always occur at the same time (Fischer et al. 2023). In addition, differences among investigators in species detection, identification, and abundance estimation can introduce uncertainty into vegetation records (Morrison et al. 2024). In this study, AGB and species richness were averaged across all quadrats within each plot, and the climatic variables were matched to the site and survey year. These procedures reduced some within‐plot sampling variation and the temporal mismatch between vegetation and climate data, but they could not completely remove methodological heterogeneity among studies. Future studies should use standardized sampling protocols and explicitly account for quadrat area, sampling effort, survey year, and data source in hierarchical models or sensitivity analyses.

Second, the positive relationship between AGB and species richness at the national scale should also be interpreted with caution. Although most formation‐level relationships were linear, the *Leymus chinensis* meadow steppe and *Kobresia pygmaea* alpine meadow formations showed unimodal responses. These results indicate that the form of the AGB–richness relationship depends on the study scale and plant formation. A global analysis of natural grasslands also found that climatic resources altered both the shape and strength of productivity–richness relationships from local to regional scales (Du et al. 2022). At low AGB, strong limitations of water, nutrients, or temperature may restrict plant establishment and result in low species richness. As resource availability increases, facilitation and complementary resource use may allow more species to coexist, resulting in an initially positive relationship. However, at high AGB, fast‐growing dominant species may capture more light and consume more soil water and nutrients, thereby increasing competitive exclusion and reducing species richness. Experimental studies have shown that competition for light is an important mechanism causing diversity loss in productive grasslands (Eskelinen et al. 2022), while increased biomass after nitrogen addition and warming can further reduce species richness through water limitation (Li, Chen, et al. 2024). A recent global study also showed that the relationship between plant diversity and biomass changed from positive under low soil nitrogen to negative under intermediate soil nitrogen (Spohn et al. 2025). Therefore, the national‐scale positive relationship found in this study should be regarded as an overall pattern within the observed AGB range rather than a universal linear response.

Third, this meta‐analysis was based on published studies identified in Web of Science and CNKI. Because the database comprised plot‐level observations rather than study‐level effect sizes with sampling variances, funnel plots and Egger's regression were not applicable. Publication‐grouped validation and publication‐cluster bootstrap resampling reduced within‐publication dependence but could not test or eliminate selective‐publication bias. Residual bias associated with database coverage, language, and selective reporting may therefore remain.

## 5 Conclusions

Using published field surveys conducted from 1982 to 2019, we evaluated how anthropogenic disturbance and climate were associated with AGB, species richness, and their relationship across Chinese grasslands. Disturbance was more important than the climatic predictors for AGB, which was greater under mowing and fencing than under grazing, declined with grazing intensity, and increased with precipitation. Species richness was influenced most strongly by MAT and was highest near −2°C to 0°C. At the national scale, AGB and species richness were positively related overall. Mowing and heavy grazing weakened the positive AGB–species richness relationship, whereas it remained strong under moderate grazing.

As the frequency and intensity of extreme drought events may increase in the future (Huang et al. 2016; Reichstein et al. 2013), grassland management should be adaptive rather than based on a fixed prescription (Li et al. 2018). Stocking rates should be matched to annual forage production and reduced during hot or dry years, because heavy grazing reduced both AGB and species richness, whereas moderate grazing maintained a stronger positive AGB–species richness relationship. Fencing may facilitate short‐ to medium‐term recovery of degraded grasslands, but its duration should be evaluated periodically because prolonged exclusion can eventually reduce AGB and species richness through litter accumulation and nutrient limitation (Liu et al. 2019). Mowing should be applied at moderate frequency after peak plant growth, with sufficient residual stubble, and should be reduced or suspended during drought and early recovery. Future grazing, fencing, and mowing regimes should therefore be adjusted according to vegetation condition, forage production, and seasonal water availability.

## Author Contributions

**Wenjie Huang:** conceptualization (equal), formal analysis (equal), methodology (equal), writing – original draft (lead). **Yuanyuan Yin:** funding acquisition (equal), visualization (equal). **Wei Wang:** conceptualization (equal), funding acquisition (equal), writing – review and editing (supporting). **Fangzheng Liu:** data curation (equal), software (equal). **Yilei Zhao:** data curation (equal). **Junsheng Li:** funding acquisition (equal), supervision (equal), writing – review and editing (equal).

## Funding

This work was supported by the National Natural Science Foundation of China (No. 42377461), the National Key Research and Development Program of China (Grant No. 2022YFF1301405), and the Hulunbuir Grassland Ecological Restoration Comprehensive Survey Project (No. DD20230474).

## Conflicts of Interest

The authors declare no conflicts of interest.

## Supporting information


**Figure S1:** Literature identification, study selection, and data‐screening workflow for the meta‐analysis of plot‐level observations from Chinese grasslands. AGB, aboveground biomass; CNKI, China National Knowledge Infrastructure. The period 1982–2019 refers to vegetation surveys rather than the database‐search window. Database‐specific hit counts and stage‐specific exclusion tallies were not retained in the original compilation.

**Table S1:** Grassland above‐ground biomass and species richness data from meta‐analysis.

## Data Availability

The data that support the findings of this study are available in the Supporting Information—S1 of this article.
