# Supplementary material for: Effects of Climatic Factors and Disturbance on Plant Above‐Ground Biomass and Species Diversity of Chinese Grasslands: A Meta‐Analysis
Source: Ecol Evol. 2026 Sep 8;16(9):e74320. doi: 10.1002/ece3.74320 (PMC13553281; doi:10.1002/ece3.74320)
Supplement: Supplementary file 1 [file ECE3-16-e74320-s001.docx]

**Supplementary Figure**

**Figure S1** Literature identification, study selection, and data-screening workflow for the meta-analysis of plot-level observations from Chinese grasslands. AGB, aboveground biomass; CNKI, China National Knowledge Infrastructure. The period 1982–2019 refers to vegetation surveys rather than the database-search window. Database-specific hit counts and stage-specific exclusion tallies were not retained in the original compilation.
